# In Vivo Matrigel Plug Assay as a Potent Method to Investigate Specific Individual Contribution of Angiogenesis to Blood Flow Recovery in Mice

**DOI:** 10.3390/ijms22168909

**Published:** 2021-08-18

**Authors:** Zeen Aref, Paul H. A. Quax

**Affiliations:** Department of Surgery, Einthoven Laboratory for Experimental Vascular Medicine, Leiden University Medical Center, 2300 RC Leiden, The Netherlands; z.aref@lumc.nl

**Keywords:** angiogenesis, arteriogenesis, animal model, Matrigel plug assay

## Abstract

Neovascularization restores blood flow recovery after ischemia in peripheral arterial disease. The main two components of neovascularization are angiogenesis and arteriogenesis. Both of these processes contribute to functional improvements of blood flow after occlusion. However, discriminating between the specific contribution of each process is difficult. A frequently used model for investigating neovascularization is the murine hind limb ischemia model (HLI). With this model, it is difficult to determine the role of angiogenesis, because usually the timing for the sacrifice of the mice is chosen to be optimal for the analysis of arteriogenesis. More importantly, the occurring angiogenesis in the distal calf muscles is probably affected by the proximally occurring arteriogenesis. Therefore, to understand and subsequently intervene in the process of angiogenesis, a model is needed which investigates angiogenesis without the influence of arteriogenesis. In this study we evaluated the in vivo Matrigel plug assay in genetic deficient mice to investigate angiogenesis. Mice deficient for *interferon regulatory factor (IRF)3, IRF7, RadioProtective 105 (RP105), Chemokine CC receptor CCR7,* and *p300/CBP-associated factor (PCAF)* underwent the in vivo Matrigel model. Histological analysis of the Matrigel plugs showed an increased angiogenesis in mice deficient of *IRF3, IRF7*, and *RP105*, and a decreased angiogenesis in *PCAF* deficient mice. Our results also suggest an involvement of *CCR7* in angiogenesis. Comparing our results with results of the HLI model found in the literature suggests that the in vivo Matrigel plug assay is superior in evaluating the angiogenic response after ischemia.

## 1. Introduction

The introduction of peripheral arterial disease (PAD) is a result of narrowing and frequently occlusion of the peripheral arteries by atherosclerotic plaque progression, which leads to impaired blood flow and subsequently ischemia in the tissue. The impaired blood flow leads to intermittent claudication and, in more severe stages when occlusion occurs, to critical limb ischemia (CLI). The prevalence of PAD increases with age to 20% in people over 70 years [1]. Current therapies for PAD are exercise rehabilitation and in severe cases endovascular revascularization or bypass surgery. Therapeutic neovascularization is a promising technique that has the potential to become an addition to conventional therapies [2].

Neovascularization is the natural mechanism that restores blood flow and recovers tissue perfusion after ischemia. The main two components of neovascularization are angiogenesis and arteriogenesis, both these mechanisms are essential for the restoration of blood flow after arterial occlusions. Identifying mediators that influence neovascularization may lead to discovering targets that can be utilized as therapeutic targets. 

Angiogenesis is the process of sprouting of endothelial cells from pre-existing blood vessels resulting in a new capillary bed and is affected by several stimulators [3]. In PAD angiogenesis provides distribution of blood to ischemic distal tissue where gangrene occurs and is essential for protecting tissue from ischemia and for tissue repair. Angiogenesis is a complex process and different cascades are involved, among which are specific angiogenic growth factors, inflammation, and epigenetic factors [4]. 

The other component of neovascularization is arteriogenesis. Arteriogenesis is an inflammation driven process induced by shear stress and leads to the maturation of the pre-existing arterioles in functioning collateral arteries [5]. 

It is essential to consider that angiogenesis and arteriogenesis are different processes. Both of these processes occur in PAD and contribute to functional improvements of blood flow after occlusion, however, discriminating between the specific contribution of each process is difficult. A frequently used in vivo model for investigation of neovascularization is the mouse hind limb ischemia (HLI) model [6]. In this model the iliac or femoral artery is occluded by ligation, most commonly the femoral artery. In general, the occlusion of the femoral artery results in arteriogenesis, also referred to as collateral formation, proximally in the thigh, and angiogenesis in the distal part of the limb. However, ligation at different anatomical levels of the iliac and femoral artery triggers different pathways of neovascularization [6]. If the ligation of the femoral artery is distal to the origin of the collateral branches, arteriogenesis will occur, as it occurs after increasing shear stress in the pre-existing arterioles proximal to the collateral arteries.

Collateral formation as a result of arteriogenesis in the proximal thigh potentially influences the blood perfusion in the distal calf by resolving the ischemia that drives the angiogenesis. This impairs the reliability of angiogenesis determination in the distal calf muscles, which is usually determined in the soleus and gastrocnemius muscles by evaluating capillary formation via CD31^+^ staining. Additionally, the timing of sacrificing the mice for histological analysis is complex, since usually this is done around two to four weeks after inducing limb ischemia. This is the optimal timing for analysis of arteriogenesis, but at this point the angiogenic response has passed its peak since the ischemia has been at least partly resolved. Therefore, to understand and subsequently intervene in the process of angiogenesis, a model is needed which investigates angiogenesis without the influence of arteriogenesis.

A suitable model to investigate angiogenesis is the in vivo Matrigel plug assay. In this assay, Matrigel is injected into the flank of mice inducing angiogenesis within the plug. The resulting angiogenesis can be evaluated after extracting the Matrigel plug. The Matrigel solution is a basement membrane preparation which is extracted from the Engelbreth–Holm–Swarm (EHS) mouse sarcoma, a tumor rich in extracellular matrix proteins [7]. It contains predominantly laminin, collagen IV, entactin, and small amounts of various growth factors. Matrigel is liquid at 4 °C and becomes a solid gel plug at 37 °C, the body temperature of mice. Matrigel mimics the physiological cell matrix and is the most frequently used substrate to investigate in vitro and in vivo angiogenesis [8]. Since the Matrigel plug is avascular at the beginning of the experiment, any vessel that is formed can be considered the result of angiogenesis. There are two main approaches in studying angiogenesis using the Matrigel plug assay. The first and most widely used approach is the use for evaluating of pro- or anti-angiogenic factors [9]. This is done by mixing a potential pro- or anti-angiogenic factor, cells, or exosomes with the Matrigel and thereafter injecting the mixture in (wild type) mice and subsequently analyzing, after the proper incubation period, the number of angiogenic vessels present in the plugs. 

In the second approach, as used in our study, unmodified Matrigel is injected it in the flank of the (genetically modified) mice. Not manipulating the gel rules out the risk of influencing its functionality and warrants consistent results. This method analyzes the influence of the factors that are altered in the mice, e.g., by genetic modification, on angiogenesis. 

The aim of this study is to demonstrate the potency of the in vivo Matrigel plug assay to investigate the role of different genetic factors in angiogenesis. To this end we performed the assay in mice genetically deficient for inflammatory factors like *interferon regulatory factor (IRF)3*, *IRF7*, *RadioProtective 105 (RP105)*, *Chemokine CC receptor CCR7*, and *p300/CBP-associated factor (PCAF*). Similar mice were used previously by our group in studies on neovascularization by means of the HLI model [10,11,12,13]. Comparing the results of current and previous research in the aforementioned genetically deficient mice will give an insight into the additional value of the Matrigel plug over the HLI model and more specifically into the specific contribution of angiogenesis on blood flow recovery in vivo. 

## 2. Results

### 2.1. Matrigel Ingrowth in Mice Deficient for Inflammation-Related Factors; IRF3^−/−^, IRF7^−/−^, and RP105^−/−^ Mice

#### 2.1.1. Increased Angiogenesis in the *IRF3^−/−^* Mice 

**Figure 1:**
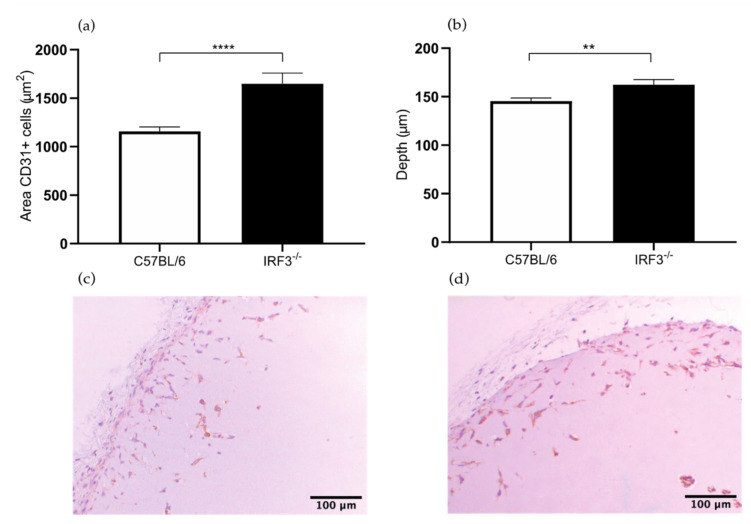
(**a**) Area of CD31^+^ cells in the subcutaneously injected Matrigel, in interferon regulatory factor (IRF)3^−/−^ and wild type C57BL/6 mice. (**b**) Quantification of the depth (μm) of ingrowth of CD31^+^ cells into subcutaneously injected Matrigel, in *IRF3*^−/−^ and wild type C57BL/6 mice. (**c**) Immunohistochemical staining of paraffin-embedded Matrigel plug of C57BL/6 7 days after Matrigel injection, using anti-CD31 antibodies. (**d**) Immunohistochemical staining of paraffin-embedded Matrigel plug of *IRF3*^−/−^ mice 7 days after Matrigel injection, using anti-CD31 antibodies. N = 6 mice per group, 2 Matrigel plugs per mouse. Values are presented as the mean SEM. ** *p* ≤ 0.01, **** *p* ≤ 0.0001.

The areas of the CD31^+^ endothelial cells in the Matrigel sections of the *IRF3^−/−^* mice were 43% larger compared to the control wild type mice (1649 versus 1157 μm^2^, *p* value < 0.0001) (Figure 1a). The depth of ingrowth of the CD31^+^ endothelial cells into the subcutaneously injected Matrigel was 11% deeper in the *IRF3*^−/−^ mice compared to the control wild type mice (162 versus 146 μm, *p* value 0.0049) (Figure 1b). This leads to the conclusion that angiogenesis was increased in the *IRF3* deficient mice, thus *IRF3* leads to decreasing angiogenesis. 

These results support the predictions made in the literature, as *IRF3* deficiency probably leads to increased angiogenesis through reduced production of type I IFNs. IRFs are transcription factors that form a dimer and translocate to the nucleus. In the nucleus, the IRFs bind to the promoter of the interferon (IFN) gene, resulting in the production of type I interferons. Type I interferons cause an increased production of several anti-angiogenic mediators such as TIMPs and a reduced production of proangiogenic factors such as VEGF, resulting in an anti-angiogenic effect. In addition, IFNs inhibit endothelial cell proliferation and migration, which are both required for angiogenesis. 

In contrast to our observations, the angiogenic response in *IRF3*^−/−^ mice which underwent HLI was decreased compared to control C57BL/6 mice [12]. We presume that the conflicting results in the HLI model are caused by the time point of evaluation. In HLI treated mice angiogenesis was assessed at sacrifice by determination of the number of CD31^+^ capillaries in both the left ischemic and right non-ischemic soleus muscle of *IRF3*^−/−^ and C57BL/6 mice. However, these data were obtained 28 days after inducing limb ischemia via surgical ligation of the femoral artery. The timing of 28 days after inducting of limb ischemia is probably not optimal for determination of the angiogenic response. At this time point angiogenesis has already passed its peak since the ischemia is partly or even totally is resolved due to concomitant arteriogenic response. In the Matrigel plug assay model analysis is performed at 7 days after injection, in a setting with full ischemia, stimulating the angiogenic activity.

#### 2.1.2. Increased Angiogenesis in *IRF7*^−/−^ Mice

**Figure 2:**
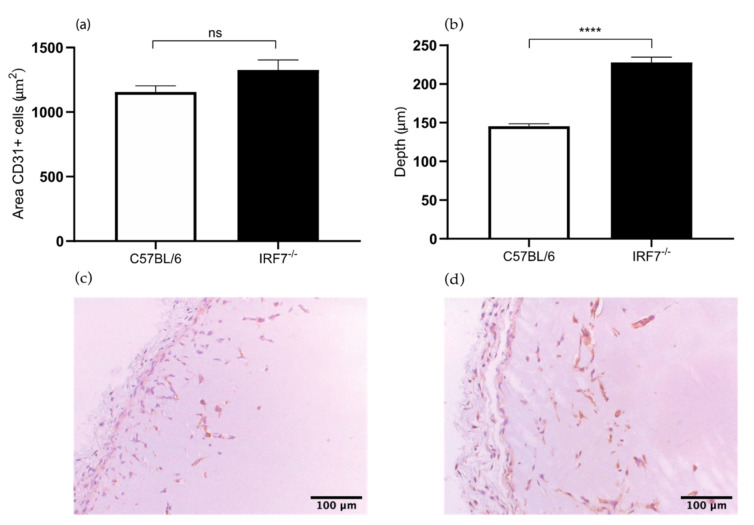
(**a**) Area of CD31^+^ cells in the subcutaneously injected Matrigel, in *IRF7*^−/−^ and wild type C57BL/6 mice. (**b**) Quantification of the depth (μm) of ingrowth of CD31^+^ cells into subcutaneously injected Matrigel, in *IRF7*^−/−^ and wild type C57BL/6 mice. (**c**) Immunohistochemical staining of paraffin-embedded Matrigel plug of C57BL/6 7 days after Matrigel injection, using anti-CD31 antibodies. (**d**) Immunohistochemical staining of paraffin-embedded Matrigel plug of *IRF7*^−/−^ mice 7 days after Matrigel injection, using anti-CD31 antibodies. N = 6 mice per group, 2 Matrigel plugs per mouse. Values are presented as the mean SEM. ns not significant. **** *p* ≤ 0.0001.

In the Matrigel sections of *IRF7*^−/−^ mice the area of the CD31+ endothelial cells were 15% larger compared to the control C57BL/6 mice. However the difference was not significant (1326 versus 1157 μm^2^, *p* value 0.07) (Figure 2a). Furthermore, the depth of ingrowth of these cells in the Matrigel plug was 56% deeper in the *IRF7*^−/−^ mice compared to control wild type mice (228 versus 146 μm, *p* value < 0.0001) (Figure 2b). These results suggest that *IRF7* is involved in the process of angiogenesis. Our experiment corroborates the hypothesis that an *IRF3* and *IRF7* deficiency leads to increased angiogenesis through reduced production of type I IFNs, as described above. In the HLI model the angiogenic response is decreased in the *IRF7*^−/−^ mice compared to C57BL/6 mice [12]. Interesting to observe is that the results of Matrigel plug assay in *IRF7*^−/−^ mice as in *IRF3*^−/−^ mice do not match the results of the HLI mouse model. An explanation for the difference is the aforementioned limitations of performing the angiogenic response analysis at 28 days after inducing HLI, which is not an optimal timing for the determination of angiogenic response.

#### 2.1.3. Increased Angiogenesis in the Matrigel Plug Assay in the *RP105^−/−^* Mice

**Figure 3:**
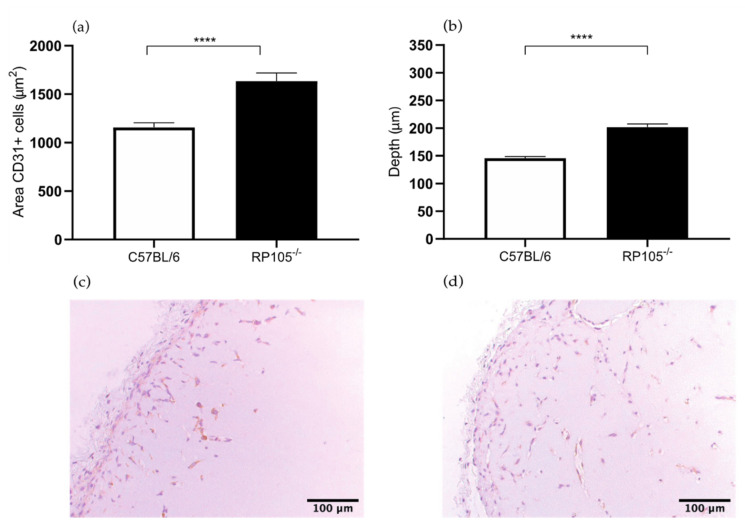
(**a**) Area of CD31^+^ cells in the subcutaneously injected Matrigel, in *RadioProtective 105 (RP105) ^−/−^* and wild type C57BL/6 mice. (**b**) Quantification of the depth (μm) of ingrowth of CD31^+^ cells into subcutaneously injected Matrigel, in *RP105^−/−^* and wild type C57BL/6 mice. (**c**) Immunohistochemical staining of paraffin-embedded Matrigel plug of C57BL/6 7 days after Matrigel injection, using anti-CD31 antibodies. (**d**) Immunohistochemical staining of paraffin-embedded Matrigel plug of *RP105^−/−^* mice 7 days after Matrigel injection, using anti-CD31 antibodies. N = 6 mice per group, 2 Matrigel plugs per mouse. Values are presented as the mean SEM. **** *p* ≤ 0.0001.

In the Matrigel plug sections of *RadioProtective 105 (RP105)* deficient mice showed a 41% larger area of CD31+ endothelial cells within the Matrigel plug in comparison to the control wild type mice (1634 versus 1157 μm^2^, *p* value < 0.0001) (Figure 3a). The depth of the cells in the Matrigel plug was 38% deeper in the *RP105^−/−^* mice compared to the control group (202 versus 146 μm, *p* value < 0.0001) (Figure 3b). These results confirm the hypothesis that RP105 is potentially an angiogenic inhibitor by inhibiting TLR4-signaling and thereby decreasing the production of several proangiogenic mediators. RP105 is a TLR4-signaling modulator and is a specific inhibitor of the TLR4-triggered response [14]. TLR4 may promote angiogenesis in pancreatic cancer tissues via activating the PI3K/AKT signaling pathway to induce VEGF expression [15]. TLR4-mediated responses also contribute to the oxygen-induced neovascularization in ischemic neural tissue [16]. However, the exact role of RP105 in angiogenesis in peripheral arterial disease remains unclear.

Previously, Bastiaansen et al. investigated angiogenesis in the HLI model by determination of capillary density in the ischemic calf muscle [11]. In their experiment the capillary density marginally increased in the gastrocnemius muscle in *RP105^−/−^* mice. However, the difference in angiogenic response measured by capillary density and size in the gastrocnemius muscle was not significant [11]. The results of the Matrigel plug assay is more evincive than the results of the HLI model, whereby *RP105* deficiency leads to increased angiogenic response 7 days after plug placement. This corresponds with our hypothesis that RP105 is potentially an angiogenic inhibitor.

### 2.2. The Role of Chemokine CC Receptor CCR7 in Angiogenesis

Previously we studied the effects of *CCR7* deficiency on blood flow recovery after HLI in *CCR7^−/−^* mice. These mice were bred on a *C57BL/6/LDLR^−/−^* background and, therefore, we used *C57BL/6/LDLR^−/−^* mice as controls [13]. Here we study the effects in angiogenesis in the Matrigel plugs in these mice. 

**Figure 4:**
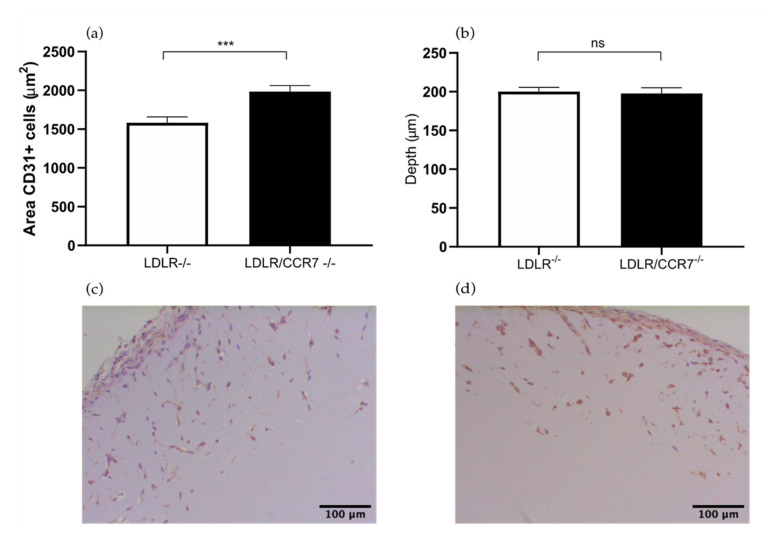
(**a**) Area of CD31^+^ cells in the subcutaneously injected Matrigel, in *LDLR^−/−^* and *LDLR^−/−^/CCR7^−/−^* mice. (**b**) Quantification of the depth (μm) of ingrowth of CD31^+^ cells into subcutaneously injected Matrigel, in *LDLR^−/−^* and *LDLR^−/−^/CCR7^−/−^* mice. (**c**) Immunohistochemical staining of paraffin-embedded Matrigel plug of *LDLR^−/−^/CCR7^−/−^* mice using anti-CD31 antibodies. (**d**) Immunohistochemical staining of paraffin-embedded Matrigel plug of LDLR^−/−^ mice using anti-CD31 antibodies. N = 7 mice per group, 2 Matrigel plugs per mouse. Values are presented as the mean SEM. *** *p* ≤ 0.001. ns = not significant.

The area of the CD31^+^ cells was 25% larger in the *LDLR^−/−^/CCR7^−/−^* mice compared to the control group (1984 versus 1583 μm^2^, *p* value 0.0003) (Figure 4a). However, the *LDLR^−/−^/CCR7^−/−^* and *LDLR^−/−^* mice showed the same depth of ingrowth of CD31^+^ cells in the Matrigel plug (198 versus 200 μm) (Figure 4b). Both the total area of endothelial cells and the depth of the ingrowth are both parameters to assess the angiogenesis in the in vivo Matrigel plug assay. However, the area of cells is a more prominent parameter, the combination of these two parameters makes it possible to make a solid conclusion. This suggests that CCR7 is involved in angiogenesis.

CCR7 is expressed by various immune cells and is involved in homing of T cells and dendritic cells to lymph nodes [17]. Furthermore, in the literature it is demonstrated that CCR7 is overexpressed in different malignant cells, which leads to the suggestion that CCR7 induces angiogenesis. However the evidence for the latter is limited [18,19]. Regarding the neovascularization stimulatory effect, the chemokine contributes to arteriogenesis via inflammatory-mediated mechanisms [20]. On the contrary, in the HLI model in *LDLR^−/−^/CCR7^−/−^* mice it was shown that the number of CD31^+^ capillaries in the gastrocnemius muscles was not significantly different from the control *LDLR^−/−^* mice [13]. In this set-up of the HLI model, the evaluation was performed 10 days after HLI, which is a good time point to investigate angiogenesis, in contrast to 28 days.

### 2.3. PCAF Deficiency Leads to Decrease in Angiogenesis

**Figure 5:**
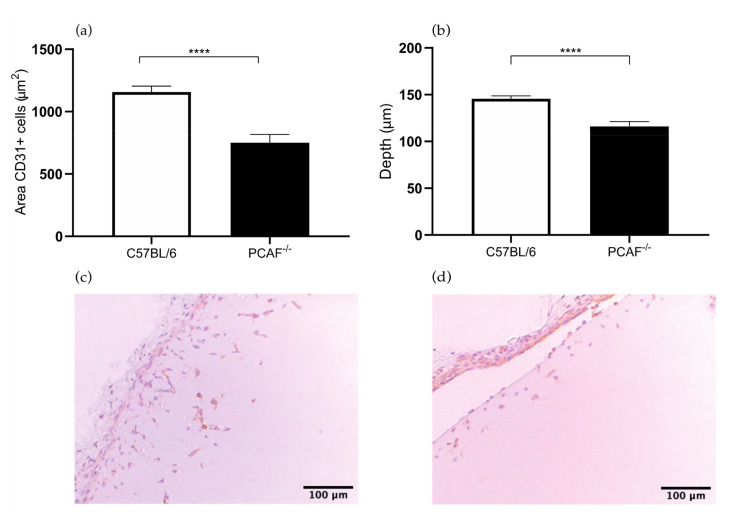
(**a**) Area of CD31+ cells in the subcutaneously injected Matrigel, in *p300/CBP-associated factor (PCAF)^−/−^* and wild type C57BL/6 mice. (**b**) Quantification of the depth (μm) of ingrowth of CD31^+^ cells into subcutaneously injected Matrigel, in *PCAF^−/−^* and wild type C57BL/6 mice. (**c**) Immunohistochemical staining of paraffin-embedded Matrigel plug of C57BL/6 7 days after Matrigel injection, using anti-CD31 antibodies. (**d**) Immunohistochemical staining of paraffin-embedded Matrigel plug of *PCAF^−/−^* mice 7 days after Matrigel injection, using anti-CD31 antibodies. N = 6 mice per group, 2 Matrigel plugs per mouse. Values are presented as the mean SEM. **** *p* ≤ 0.000.

In the *PCAF*^−/−^ Matrigel sections, the area of the CD31^+^ endothelial cell area was 35% smaller compared to the control group (751 versus 1157 μm^2^, *p* value < 0.0001) (Figure 5a). The depth of the CD31^+^ endothelial cells in the Matrigel plug was 21% less deep in the *PCAF^−/−^* mice compared to the control group (116 versus 146 μm, *p* value < 0.0001) (Figure 5b). The results of the Matrigel plug assay in *PCAF^−/−^* mice demonstrate that PCAF has a role in angiogenesis. This result confirms the hypothesis that PCAF may have a role in angiogenesis considering the essential role of HIF-1 in angiogenesis. P300/CBP-associated factor (PCAF) acetylates histones H3 and H4 and this histone acetylating activity is crucial for NF-KB-mediated gene transcription and regulates inflammation-related genes [21]. PCAF also mediates the regulation of hypoxia-inducible factor-1α (HIF-1α), which increases lysyl-acetylted HIF-1α and delays the PHD-independent degradation of HIF-1α [22]. HIF-1 regulates the expression of proangiogenic factors and is even a master stimulator of vascular endothelial growth factor [23]. Previously, our group investigated the involvement of PCAF in arteriogenesis as is a key regulator of this process [10]. Additionally, our group showed that PCAF regulates vascular inflammation [24].

As to angiogenesis in hind limb ischemia, it has not yet been investigated in *PCAF^−/−^* mice. In consideration that PCAF mediates the regulation of hypoxia-inducible factor-1α (HIF-1α) and HIF-1 regulates the expression of proangiogenic factors, such as vascular endothelial growth factor, this is the result of this study that we expected.

## 3. Discussion

In this study we were able to quantify the angiogenic response in Matrigel plugs of various genetically modified mouse strains and compare these results with the results obtained in the HLI model. We found that the angiogenic response in the same genetic deficient mice determined through the Matrigel plug assay could be different from the angiogenic results found in the HLI model. The results showed that the in vivo Matrigel plus assay is more reliable than the HLI model to the determine the angiogenic response. 

We performed the in vivo Matrigel plug assay in *IRF3*^−/−^ and *IRF7*^−/−^ mice because the exact role of these components is unknown and theoretically *IRF3* and *IRF7* may be anti-angiogenic and has a potential in therapeutic angiogenesis. The results of the Matrigel plug model in *IRF3*^−/−^ and *IRF7*^−/−^ mice were that angiogenesis is increased in *IRF3*^−/−^ and *IRF7*^−/−^ mice. These results are the opposite to the results of the HLI model, where it was demonstrated that the angiogenic response decreased in the *IRF3*^−/−^ and *IRF7*^−/−^ mice compared to C57BL/6 mice [12]. Since in the HLI model, the evaluation of angiogenesis was performed 28 days after surgery and the angiogenesis in the distal calf muscles is influenced by arteriogenesis proximal, we believe that the results in Matrigel plug assay are more reliable. 

Mice deficient in *RP105* show a severely impaired blood flow recovery after HLI, where arteriogenesis was reduced [11], whereas the effects on angiogenesis were not studied. The Matrigel plug assay data from *RP105^−/−^* mice showed that *RP105* deficiency leads to increased angiogenesis. Previously we have shown that CCR7 expression is rapidly upregulated after induction of HLI in mice, and the neovascularization response after HLI in *LDLR^−/−^/CCR7^−/−^* was reduced due to effects on arteriogenesis as well as angiogenesis [13]. Our data strongly support the involvement of CCR7 in the angiogenesis response, next to its role in arteriogenesis. Along the same line, we previously demonstrated a hampered blood flow recovery in *PCAF^−/−^* mice due to a decreased arteriogenesis [10] without analyzing the angiogenesis response. Here we show, using the Matrigel plug assay in *PCAF^−/−^* mice, that PCAF has a role in angiogenesis and more study to elucidate the role of PCAF in angiogenesis is needed. 

In the in vivo Matrigel plug assay for accurate quantification through histological analysis, numerous tissue slides for each plug are needed. This makes it a time-consuming process. However, the histological analysis is still the preferred method to study angiogenesis in Matrigel plug assay as it provides information on morphology and localization of the endothelial cells, which cannot be obtained through other techniques. In histological analysis we used the area of endothelial cells and the depth of the ingrowth as a result parameter. It is challenging to interpret the results if both parameters do not show the same result. In our experiments we used a combination of both parameters and considered the area of the cells as a superior parameter. 

Alternative quantitative techniques for the assessment of angiogenesis in the in vivo Matrigel plug model are hemoglobin content determination, injecting dextran, Matrigel cytometry, or using the qPCR technique [9,25,26,27]. The hemoglobin content assay is used to assess the blood content in the newly formed vessels. However this assay cannot differentiate between stagnant blood, blood in the capillaries, larger vessels, or in the vessels in the surrounding granulation tissue [28,29]. Strict separation of the surrounding tissue from the edge of the plug is challenging and can lead to damaging the edge where the most angiogenesis occurs. The other alternative method is injecting the dextran into the tail vein and extracting it from the plug for quantification. This approach can also not differentiate between the presence of stagnant blood due hemorrhage and blood in the vessels, also non-perfused vessels due to compression at the time of harvesting is not quantified. The new method is isolating RNA from the plugs and use qPCR of EC genes as a quantification technique [27]. However, the limited cellularity in the control plugs lead to a low yield of total RNA, making the comparison between two sets of samples unreliable. 

## 4. Materials and Methods

### 4.1. Mice

All animal experiments were performed in compliance with Dutch government guidelines and the Directive 2010/63/EU of the European Parliament, all experiments were approved by the animal welfare committee of the LUMC under approval code 12173 (19-11-2012). In this study we used mice deficient for *IRF3*, *IRF7*, *RP105* and *PCAF* [10,11,12]. Also, *LDLR^−/−^/CCR7^−/−^* and *LDLR*^−/−^ mice were used [13]. All strains have a C57BL/6 background. In general, we used n= 6 mice per group. Wild type C57BL/6 were used as control. The mice were used at the age of 10–14 weeks.

### 4.2. The In Vivo Matrigel Plug Assay 

**Figure 6:**
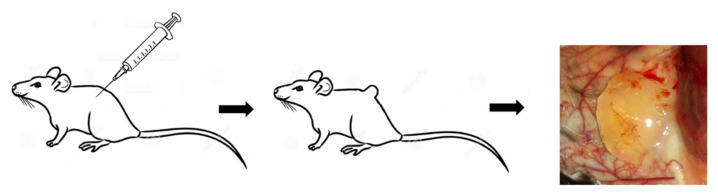
Matrigel plug in the flank of a mouse.

A total of 500 μL of Matrigel Solution (BD Biosciences, Vianen, the Netherlands) was injected subcutaneously in the dorsal side of the mice, both the left and right flank. The solution had a temperature of 4 °C at time of injection, forming a plug as it warmed up to body temperature (37 °C) [8]. After seven days the mice were sacrificed and the Matrigel plug and the surrounding granulation tissue were removed. The color of the viscous plugs ranged from a (light) yellow (Figure 6), to a pink or red depending on the amount of blood vessel ingrowth. The Matrigel plugs were fixed in formaldehyde, embedded into paraffin blocks, and sectioned into slides of 5 µm.

### 4.3. CD31-Immunohistochemistry Staining

The paraffin-embedded sections of the Matrigel plug (5 µm) were used for histological analysis. Sections were stained using anti-CD31 antibodies (BD Biosciences). The CD31-immunohistochemistry staining was performed to detect the CD31^+^ endothelial cells in the Matrigel plugs. Most angiogenesis is observed at the edge of the plug, with ingrowth toward the center of the plug.

**Figure 7:**
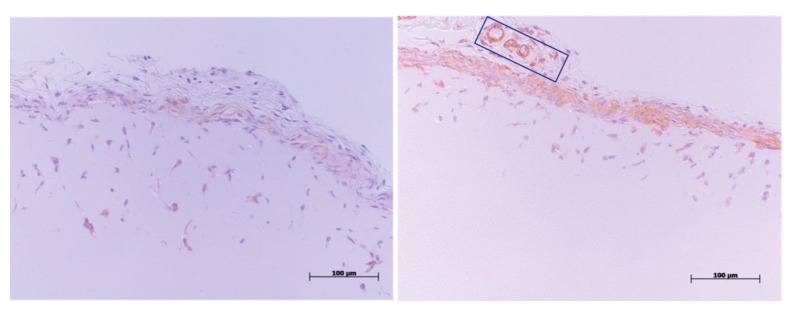
Representative images of the CD31 immunohistochemistry staining. CD31 positive cells (i.e., endothelial cells) are stained and visible as a brown staining. In the right figure, a blood vessel in the membrane surrounding the Matrigel plug is stained and serves as an internal positive control. In the box in the right panel some CD31 positive vessel structures in the surrounding granulation tissue around the plug can been seen, demonstrating the specificity of the endothelial cell staining. The left panel is representative of a section with a deeper ingrowth of endothelial cells. The maximal depth to the ingrowth as well as the CD31+ area in the plug were quantified using Image J software.

Photomicrographs of the CD31-stained sections were made and morphometric image analysis was performed (Figure 7). The Matrigel area (μm^2^), endothelial cell areas (μm^2^), endothelial cell ingrowth (%), and maximal endothelial cell depth (μm) were measured for quantification of angiogenesis using Image J software.

### 4.4. Statistical Analysis

Results are presented as mean ± SEM. Comparisons between groups were performed using Student *t*-test or Mann–Whitney U-test. Statistical analyses were performed using GraphPad Prism 7. A *p* value of <0.05 was considered statistically significant.

## 5. Conclusions

In conclusion, the Matrigel plug assay in mice should be the method of choice for the in vivo evaluation of angiogenesis and has an added value over HLI model in the research of neovascularization. The in vivo Matrigel plug assay can be used to identify factors that are involved in angiogenesis. This study indicates that RP105, IRF3, IRF7, CCR7, and PCAF are involved in angiogenesis.

## Data Availability

All relevant data will be made available upon request.

## References

[1] Norgren L., Hiatt W.R., Dormandy J.A., Nehler M.R., Harris K.A., Fowkes F.G., Rutherford R.B. (2007). Inter-society consensus for the management of peripheral arterial disease. Int. Angiol. J. Int. Union Angiol..

[2] Raval Z., Losordo D.W. (2013). Cell therapy of peripheral arterial disease: From experimental findings to clinical trials. Circ. Res..

[3] Carmeliet P. (2003). Angiogenesis in health and disease. Nat. Med..

[4] Potente M., Gerhardt H., Carmeliet P. (2011). Basic and therapeutic aspects of angiogenesis. Cell.

[5] Heil M., Eitenmuller I., Schmitz-Rixen T., Schaper W. (2006). Arteriogenesis versus angiogenesis: Similarities and differences. J. Cell Mol. Med..

[6] Aref Z., de Vries M.R., Quax P.H.A. (2019). Variations in Surgical Procedures for Inducing Hind Limb Ischemia in Mice and the Impact of These Variations on Neovascularization Assessment. Int. J. Mol. Sci..

[7] Kleinman H.K., Martin G.R. (2005). Matrigel: Basement membrane matrix with biological activity. Semin. Cancer Biol..

[8] Nowak-Sliwinska P., Alitalo K., Allen E., Anisimov A., Aplin A.C., Auerbach R., Augustin H.G., Bates D.O., van Beijnum J.R., Bender R.H.F. (2018). Consensus guidelines for the use and interpretation of angiogenesis assays. Angiogenesis.

[9] Passaniti A., Taylor R.M., Pili R., Guo Y., Long P.V., Haney J.A., Pauly R.R., Grant D.S., Martin G.R. (1992). A simple, quantitative method for assessing angiogenesis and antiangiogenic agents using reconstituted basement membrane, heparin, and fibroblast growth factor. Lab. Investig..

[10] Bastiaansen A.J., Ewing M.M., de Boer H.C., van der Pouw Kraan T.C., de Vries M.R., Peters E.A., Welten S.M., Arens R., Moore S.M., Faber J.E. (2013). Lysine acetyltransferase PCAF is a key regulator of arteriogenesis. Arterioscler. Thromb. Vasc. Biol..

[11] Bastiaansen A.J., Karper J.C., Wezel A., de Boer H.C., Welten S.M., de Jong R.C., Peters E.A., de Vries M.R., van Oeveren-Rietdijk A.M., van Zonneveld A.J. (2014). TLR4 accessory molecule RP105 (CD180) regulates monocyte-driven arteriogenesis in a murine hind limb ischemia model. PLoS ONE.

[12] Simons K.H., de Vries M.R., de Jong R.C.M., Peters H.A.B., Jukema J.W., Quax P.H.A. (2019). IRF3 and IRF7 mediate neovascularization via inflammatory cytokines. J. Cell. Mol. Med..

[13] Nossent A.Y., Bastiaansen A.J., Peters E.A., de Vries M.R., Aref Z., Welten S.M., de Jager S.C., van der Pouw Kraan T.C., Quax P.H. (2017). CCR7-CCL19/CCL21 Axis is Essential for Effective Arteriogenesis in a Murine Model of Hindlimb Ischemia. J. Am. Heart Assoc..

[14] Yildirim C., Nieuwenhuis S., Teunissen P.F., Horrevoets A.J., van Royen N., van der Pouw Kraan T.C. (2015). Interferon-Beta, a Decisive Factor in Angiogenesis and Arteriogenesis. J. Interferon Cytokine Res..

[15] Sun Y., Wu C., Ma J., Yang Y., Man X., Wu H., Li S. (2016). Toll-like receptor 4 promotes angiogenesis in pancreatic cancer via PI3K/AKT signaling. Exp. Cell Res..

[16] He C., Sun Y., Ren X., Lin Q., Hu X., Huang X., Su S.-B., Liu Y., Liu X. (2013). Angiogenesis mediated by toll-like receptor 4 in ischemic neural tissue. Arterioscler. Thromb. Vasc. Biol..

[17] Forster R., Davalos-Misslitz A.C., Rot A. (2008). CCR7 and its ligands: Balancing immunity and tolerance. Nat. Rev. Immunol..

[18] Chi B.J., Du C.L., Fu Y.F., Zhang Y.N., Wang R.W. (2015). Silencing of CCR7 inhibits the growth, invasion and migration of prostate cancer cells induced by VEGFC. Int. J. Clin. Exp. Pathol..

[19] Xiong Y., Huang F., Li X., Chen Z., Feng D., Jiang H., Chen W., Zhang X. (2017). CCL21/CCR7 interaction promotes cellular migration and invasion via modulation of the MEK/ERK1/2 signaling pathway and correlates with lymphatic metastatic spread and poor prognosis in urinary bladder cancer. Int. J. Oncol..

[20] Shireman P.K. (2007). The chemokine system in arteriogenesis and hind limb ischemia. J. Vasc. Surg..

[21] Sheppard K.A., Rose D.W., Haque Z.K., Kurokawa R., McInerney E., Westin S., Thanos D., Rosenfeld M.G., Glass C.K., Collins T. (1999). Transcriptional activation by NF-kappaB requires multiple coactivators. Mol. Cell. Biol..

[22] Lim J.H., Lee Y.M., Chun Y.S., Chen J., Kim J.E., Park J.W. (2010). Sirtuin 1 modulates cellular responses to hypoxia by deacetylating hypoxia-inducible factor 1alpha. Mol. Cell.

[23] Zimna A., Kurpisz M. (2015). Hypoxia-Inducible Factor-1 in Physiological and Pathophysiological Angiogenesis: Applications and Therapies. BioMed Res. Int..

[24] de Jong R.C.M., Ewing M.M., de Vries M.R., Karper J.C., Bastiaansen A., Peters H.A.B., Baghana F., van den Elsen P.J., Gongora C., Jukema J.W. (2017). The epigenetic factor PCAF regulates vascular inflammation and is essential for intimal hyperplasia development. PLoS ONE.

[25] Auerbach R., Lewis R., Shinners B., Kubai L., Akhtar N. (2003). Angiogenesis assays: A critical overview. Clin. Chem..

[26] Adini A., Fainaru O., Udagawa T., Connor K.M., Folkman J., D’Amato R.J. (2009). Matrigel cytometry: A novel method for quantifying angiogenesis in vivo. J. Immunol. Methods.

[27] Coltrini D., Di Salle E., Ronca R., Belleri M., Testini C., Presta M. (2013). Matrigel plug assay: Evaluation of the angiogenic response by reverse transcription-quantitative PCR. Angiogenesis.

[28] Norrby K. (2006). In vivo models of angiogenesis. J. Cell. Mol. Med..

[29] Auerbach R., Akhtar N., Lewis R.L., Shinners B.L. (2000). Angiogenesis assays: Problems and pitfalls. Cancer Metastasis Rev..

